# Serum biomarkers for the diagnosis of chronic recurrent multifocal osteomyelitis (CRMO)

**DOI:** 10.1186/1546-0096-13-S1-O77

**Published:** 2015-09-28

**Authors:** S Hofmann, A-S Kubasch, U Range, M Laass, A Rösen-Wolff, T Schwarz, C Hofmann, H Girschick, H Morbach, C Hedrich

**Affiliations:** 1 Children's Hospital, Universitätsklinikum Carl Gustav Carus, TU Dresden, Pediatric Rheumatology and Immunology, Dresden, Germany; 2 Institute for Medical Informatics and Biometry, Universitätsklinikum Carl Gustav Carus, TU Dresden, Dresden, Germany; 3 Children's Hospital, Universitätsklinikum Carl Gustav Carus, TU Dresden, Pediatric Gastroenterology, Dresden, Germany; 4 St. Josef Stift Sendenhorst, Department of Pediatric Rheumatology, Sendenhorst, Germany; 5 Children's Hospital, University of Würzburg, Würzburg, Germany; 6 Children's Hospital, Vivantes Klinikum-Friedrichshain, Berlin, Germany; 7 Uniklinikum Dresden, Pediatrics, Dresden, Germany

## Introduction

Chronic nonbacterial osteomyelitis (CNO) is an autoinflammatory bone disorder mostly affecting children and adolescents. Chronic recurrent multifocal osteomyelitis (CRMO) is the most severe form of CNO. It is characterized by recurring episodes of bone inflammation that can last for years and may cause chronic pain, pathological fractures, and disability. Despite recent advances in targeting disease mechanisms, the exact pathophysiology of CNO/CRMO remains unknown. Diagnosis of CNO can be challenging, because symptoms tend to be mild and highly variable, and is further complicated by the absence of widely accepted diagnostic criteria and disease biomarkers.

## Objectives

The aim of our study was to determine serum biomarkers for the diagnosis of CRMO, discriminating CRMO patients from healthy individuals and patients with other inflammatory conditions (Crohn's disease and JIA).

## Methods

Serum of treatment-naïve CRMO patients was collected at the time of diagnosis (N=56). As controls, sera from treatment-naïve age matched patients with Crohn's disease (N=62) or JIA (N=27), as well as healthy individuals (N=62) were collected. Sera were subjected to proteomic analysis, using the Human Cytokine 25-plex Assay (Life Technologies) on the Luminex^^®^^ 200™ platform. Standard inflammation markers from our routine clinical chemistry laboratory (CrP) were included in our analysis. Statistical analysis was performed using non-parametric Kruskal-Wallis tests, Mann-Whitney-U tests, and canonical discriminant analysis to test between disease and control groups.

## Results

The following (9 out of 25) serum proteins were detectable and significantly differed between groups: IL-1RA, IL-2R, IL-6, IL-12, Eotaxin, MCP-1, MIG, MIP-1b, RANTES. Kruskal-Wallis and Mann-Whitney U tests confirmed significant differences between three groups: CRMO, Crohn's disease, and healthy controls. Biosamples from CRMO and JIA patients were less clearly distinguishable. Multi-component canonical discriminant analysis allowed for the definition of algorithms differentiating between CRMO, Crohn's disease, and healthy controls. We failed to differentiate sera from patients with JIA from CRMO samples. However, JIA and CRMO can usually be differentiated by their clinical presentation.

## Conclusion

Our serum marker based discrimination algorithm can discriminate CRMO patients from patients with Crohn's disease and healthy individuals. Though confirmation of our findings in larger, multi-ethnical cohorts is currently lacking, in a clinical setting this may prove useful and valuable to differentiate between individuals with “bone pain” and CRMO.

